# Local and systemic inflammation after implantation of a novel iron based porous degradable bone replacement material in sheep model

**DOI:** 10.1038/s41598-021-91296-y

**Published:** 2021-06-08

**Authors:** Bernd Wegener, Maik Behnke, Stefan Milz, Volkmar Jansson, Christian Redlich, Walter Hermanns, Christof Birkenmaier, Korbinian Pieper, Thomas Weißgärber, Peter Quadbeck

**Affiliations:** 1 Department of Orthopedic Surgery, Physical Medicine and Rehabilitation, Ludwig-Maximilians-University Munich, Marchioninistraße 15, 81377 Munich, Germany; 2 Department of Veterinary Pathology, Ludwig-Maximilians-University of Munich, Munich, Germany; 3 Veterinary Clinic Oberhaching, Oberhaching, Germany; 4 Department of Anatomy, Ludwig-Maximilians-University of Munich, Munich, Germany; 5 AO Research Institute, AO Foundation, Davos, Switzerland; 6 Department of Surgery and Genecology of Animals, Ludwig-Maximilians-University Munich, Munich, Germany; 7 Fraunhofer Institute for Manufacturing and Advanced Materials (IFAM), Dresden, Germany

**Keywords:** Tissue engineering, Biomaterials

## Abstract

Despite the high potential of healthy bone to regenerate, the reconstruction of large bone defects remains a challenge. Due to the lack of mechanical stability of existing bone substitutes, recently developed degradable metallic alloys are an interesting alternative providing higher load-bearing capabilities. Degradable iron-based alloys therefore might be an attractive innovation. To test the suitability of a newly-designed iron-based alloy for such applications, an animal experiment was performed. Porous iron-based degradable implants with two different densities and a control group were tested. The implants were positioned in the proximal tibia of Merino sheep. Over a period of 6 and 12 months, blood and histological parameters were monitored for signs of inflammation and degradation. In the histological evaluation of the implants` environment we found degraded alloy particles, but no inflammatory reaction. Iron particles were also found within the popliteal lymph nodes on both sides. The serum blood levels of phosphorus, iron and ferritin in the long term groups were elevated. Other parameters did not show any changes. Iron-based degradable porous bone replacement implants showed a good biocompatibility in this experiment. For a clinical application, however, the rate of degradation would have to be significantly increased. Biocompatibility would then have to be re-evaluated.

## Introduction

Bone grafts are the second most frequently transplanted tissue and an important treatment tool in Orthopaedic surgery and the gold standard for skeletal reconstruction in case of inadequate native bone^1–5^. Despite a high regenerative potential of healthy bone, the replacement of large bone defects following complicated fractures, non-union, tumor resections, infection-related bone loss or in revision arthroplasty are persisting challenges. Treatment of these bone defects is currently possible by several methods, but autologous bone transplantation is still considered the gold standard. Unfortunately, this procedure provides only small amounts of mechanically insufficient bone^1,6^. A great variety of biodegradable materials has been developed as bone substitutes. With an annual market volume of over 800 million U.S. dollars and with a clear upwards trend, they also have become important from an economic point of view^6^.

In principle, the human organism is capable of building new bone, but it requires adequate time and the right combination of stability and cyclical compressive loading to generate the necessary amounts of bone with satisfactory stability. Therefore, the aim is to generate implants that warrant a high primary stability, enable bone ingrowth by material-dependent slow absorption of the implant and provide gradual biological load transmission^7^. Ideally, the progressing osteointegration and implant degradation would work in an optimal bidirectional adaption to their respective consistencies. To allow for vascularization and the ingrowth of osteons, an open-porous implant structure with cavities of 100–500 μm in diameter is needed^8–10^. Due to the lack of mechanical stability of existing resorbable and degradable bone substitutes, recently developed degradable metallic alloys are an interesting alternative providing higher load bearing properties^11,12^.

An appropriate alloy for a metallic bioresorbable bone implant should degrade very slowly while the newly formed bone builds up stability and load transformation by progressive osteointegration^7^. Implant development currently focuses on iron- and magnesium-based alloys, whose mechanism of degradation is based on metal corrosion. Magnesium-based alloys are currently being tested in the fields of cardiology, cardiac surgery and orthopedic trauma surgery^13–16^. During the corrosion process of magnesium-based implants in chloride-containing body fluids, magnesium and other alloying elements are released in the form of ions accompanied by hydrogen gas release^13,17^. Witte et al. showed an increase in bone mass around the inserted magnesium implant and an accumulation of calcium phosphate using a guinea pig model^18^. The authors concluded from this finding that an increase in the osteoblast activity is induced by degradation of the alloy. Therefore, magnesium-based alloys may provide in the future provide an alternative for bone surgery but they have a higher degradation rate than the tissue healing rate^18,19^.

However, in order to take over a load-bearing function within the treatment of bone loss, a slow corrosion-related degradation rate under physiological body conditions is substantial. Here, iron-based alloys appear to present an adequate alternative, as the newly formed bone is not able to ensure load transfer in case of too rapid degradation of the implant^20^. These iron-based alloys have already demonstrated a high compressive strength and good in-vivo biocompatibility during the development of biodegradable vascular stents^21–23^. In cell culture, no apoptotic effects but only a decreased proliferation of smooth muscle cells was detected^24^. Different alloys have been examined for their electrochemical properties, degradation rate and cytotoxicity^25–27^. Out of the variety of candidate materials, iron-based alloys containing Mn, W, C und S were deemed to be suitable for the development of biodegradable bone replacement implants. To find the most appropriate metal, an alloy was designed by our own research group^28^. A preliminary *in-vitro* test was performed to evaluate degradability and cytotoxicity of iron-based compositions. An alloy of iron and 0.6 wt. % phosphorus showed the most favorable properties regarding the material´s technical workability, corrosion resistance and cell toxicity. Tests were conducted using a static monolayer-culture and a perfusion-chamber system, whose continuous flow assured efflux of potential cytotoxic agents.

In previously published parts of this study, we were able to show that degradation occurs in clinical animal experiments but does not reach the desired level for open porous implants made of iron with 0.6 wt.% of phosphorus^29^. This finding is in accordance with those by Kraus et al. who implanted pins made of Fe-10Mn-1Pd and Fe-21Mn-0.7C-1Pd into the femoral mid-diaphyseal region of rats^30^. However, there is no data on local or systemic inflammatory responses of degradable iron-based materials for orthopedic applications that we are aware of. Other recent studies on the suitability of porous iron-based bone replacement implants—mostly combined with the application of biodegradable polymers to the metal surface to enhance surface degradation—are still in the preclinical state^31–33^ or do not consider these aspects^34^.

Thus, the aim of the present complementary study is to analyze in detail if implantation of the newly developed degradable iron-based porous bone substitute material results in implant-related local or systemic inflammatory responses in experimental animals. In addition, we aimed to clarify whether degradation product deposits occur in tissues adjacent to the implant, local lymph nodes or parenchymal organs. Finally, possible changes of iron and phosphate metabolism were examined in the animals’ blood.

## Materials and methods

### Implant

**Figure 1:**
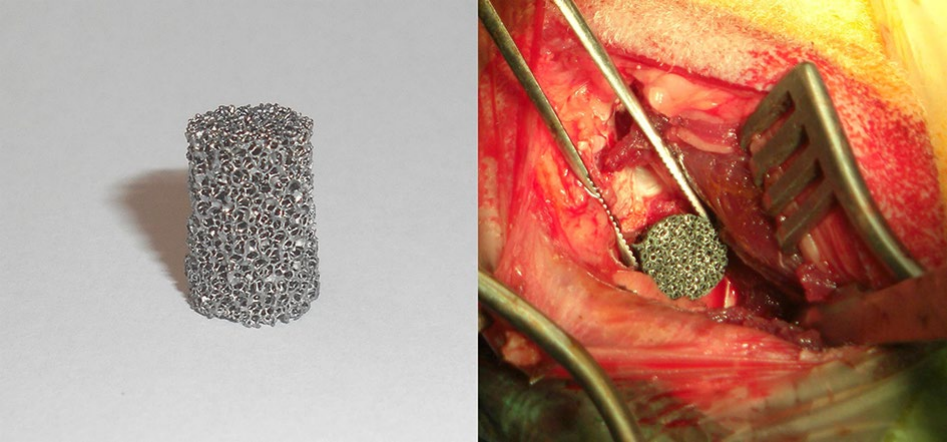
Degradable iron alloy implant and operative site of implant insertion.

In our own previous work, a degradable metal implant was developed^28,29^. The material is an open cell metal foam with a nominal cell size of 45 pores per inch, porosities of 82% and 87% and has been manufactured by means of a powder metallurgical replication method. This method involves the coating of reticulated polyurethane sponges by slurry impregnation using double rubber rollers. The cell size is 45 pores per inch (Foampartner Reisgies, Germany) which correlates to a mean cell diameter of approx. 1.2 mm. Water based slurries with PVA-binder and solids content between 87 and 90% were used. The density was adjusted by controlling the coating mass of the powder suspension. In order to produce alloys with a phosphorus contents of 0.6 wt.-%, carbonyl iron powder (BASF, Germany, mean particle size 4 µm) was mixed with Fe_3_P particles (Atmix, Japan, mean particle size 1.5 µm) in a ratio of 96.2:3.8. In the next step, the components were debinded at 500 °C in an ArH_2_-atmosphere and sintered at 1080 °C in pure hydrogen. After sintering, the basic material consists of Iron with a phosphorus contents of 0.6 wt.-% and a carbon contents of 0.24–0.36 wt.-%. In this way, open cell foam sheets (100 × 100 × 10 mm^3^) were produced. In the last step, slightly conical implants (*Ø*_*bottom*_ = 10 mm, *Ø*_*top*_ = 10.2 mm, *h* = 15 mm) were cut by waterjet cutting. To eliminate oxides caused by the water cutting process the implants were finally reduced in pure hydrogen at 800 °C^29^. The final fabricated implant and its surgical implantation is shown in Fig. 1.

### Animal experiment

The study protocol was approved by the animal testing board of the Government of Oberbayern and registered (experiment number 55.2-1-54-2531-130-07). Experiments were performed in accordance to national guidelines for animal experiments, the guidelines of the European Convention for the Protection of Vertebrate Animals used for Experimental and Other Scientific Purposes and the ARRIVE guidelines^35^ using sixty skeletally mature Merino sheep with at least 18 months of age and a minimum body weight of 60 kg. The animals were divided into 5 groups with a calculated group size of 11 animals (significance level α = 0.05 and desired power of 0.8 as a basis). Five reserve animals have been requested in case of failures (death, infection or other complications).

A bone defect was administered (diameter 10 mm, depth 15 mm). In the control group its margin and marked with commercially available surgical wires (diameter 1.4 mm) and no implants were inserted. In the experimental groups. cylindrical implants (diameter 10 mm, depth 15 mm) of two different densities (1.0 und 1.4 g/cm^3^) were placed for 6 month (short term group) or 12 months (long term group).In case of a serious medical condition (pneumonia), unstable implant bolting or occurrence of other complications animals were replaced according to the veterinary license.

At the end of the experiments, the following groups were available for evaluation: control group of 10 animals (one replacement animal, two drop outs), short term group 1.0 g/cm^3^ of 10 animals (one drop out), long term group 1.0 g/cm^3^ of 11 animals (one replacement animal, one drop out), short term group 1.4 g/cm^3^ of 14 sheep (three replacement animals), long term group 1.4 g/cm^3^ of 11 animals. In the short term group 1.4 g/cm^3^, three animals suffered from pneumonia. Sheep survive this illness rarely, so that we included the replacement animals early in the experiment. Since pneumonia could be treated successfully, so this group included 14 animals in the end.

Initially 0.5 mg/kg Meloxicam (Metacam, Boehringer Ingelheim GmbH, Germany) was administered subcutaneously. While anesthesia was induced using a combination of 0.2 mg/kg diazepam (Diazepam Desitin injection, Desitin Arzneimittel, Germany), 0.1–0.2 mg/kg xylazine (Narcoxyl injection, Veterinaria AG, Switzerland) and 15 mg/kg ketamine hydrochloride (Ketavet, Pharmacia & Upjohn GmbH, Germany), it was sustained with xylazine and ketamine. Pure oxygen was administered for the duration of the operation through a mask and antibiotic coverage conducted with amoxicillin/clavulanic acid (2.2 g/55 ml NaCl; SmithKline Beecham Pharma GmbH, Munich, Germany).

The medial tibia plateau was exposed via a longitudinal skin incision on the medial side and a defect of 10 mm in diameter by 15 mm in depth was drilled. After thorough irrigation with NaCl and debridement, the implant was inserted. The wound was closed by means of suture. Under pain control with Buprenorphin-HCl 0.324 mg (Temgesic, Essex Pharma, Munich, Germany) the animals started to load the implantation site at the second day after surgery without limping or other signs of pain^29^.

To detect changes in serum parameters for iron, phosphorus, ferritin, alkaline phosphatase, haptoglobin, hemoglobin, hematocrit, erythrocytes, platelets, leukocytes and differential count, blood samples from the external jugular vein were taken regularly at day 1 and after 1, 2, 6, 10, 16, 22, 28, 42 and 52 weeks after surgery.

The animals were euthanized 6 or 12 months after operation by intravenous administration of 20 ml Pentobarbital (Narcoren 16 g/100 ml; Merial GmbH, Hallbergmoos, Germany) and 20 ml KCl (1 M Potassium-chloride-solution, Pfrimmer Baxter GmbH, Unterschleißheim, Germany). Subsequently, autopsy of the animals was performed by a veterinary pathologist. This involved evaluation of pathological-anatomical findings and tissue sampling from the spleen, liver, heart, lungs, kidneys, brain, local lymph nodes (popliteal and iliofemoral lymph nodes of both sides), and tissue of the implant environment. A ceramic knife was used for organ preparation in order to to avoid contamination with iron particles.

### Histology

For histopathological evaluation, the following samples were taken: organ samples from spleen, liver, heart, lungs, kidneys, and brain; a cross section of the local lymph nodes (popliteal and iliofemoral lymph nodes of both sides); tissue adjacent to the implant (ca. 15 × 5 × 2.5 mm, laminated, including connective tissue, fat tissue and skeletal muscle). Formaldehyde-fixed tissues underwent routine processing, embedded in paraffin and plastic and stained with haematoxylin and eosin (H&E) according to Giemsa^36^. All pathologic changes were recorded, and particular attention was directed to inflammatory components and iron deposits. Turnbull blue staining was performed for the detection of local iron deposition in tissues adjacent to the implant and the local lymph nodes as well as for the detection of possible systemic iron accumulation in organ samples. The degree of inflammation was categorized in four levels (no, mild, moderate or severe inflammation), dependent on the quantity of inflammatory cells in the examined sections.

### Statistical evaluation

For statistical evaluation of local iron deposits and inflammatory response, non-parametric tests with a significance level of α = 0.05 were used exclusively. In order to test, whether score values of the individual organs or lymph nodes and laboratory tests originate from the same population, the Kruskal–Wallis test for k-independent samples was used. The Kruskal–Wallis test checks the null hypothesis with the statement—the location parameters are the same in all groups. Whenever significances or rejection of the null hypothesis with the Kruskal–Wallis occurred in individual pairs of parameters, these would be adressed with the Mann–Whitney test for two independent samples. To evaluate whether chronological sequence of laboratory parameters within the test period and the measured laboratory values are linked, calculation was performed using the Spearman´s rank correlation coefficient for the entire experimental group that was treated with the implant of 1.4 g/cm^3^ density.

The evaluation was performed using the statistical program SPSS 19.0 (IBM, USA).

Due to an assumed transport-related haemolysis in the first laboratory tests of the control group and the short-term implant group of 1.0 g/cm^3^, a statistical construction aid was used for the comparison between the initial laboratory analysis and the last measured value. Data groups of each initial laboratory value (long-term implant 1.4 g/cm^3^, short-term implant 1.4 g/cm^3^, and long-term implant 1.0 g/cm^3^) were analyzed using the Kruskal–Wallis test for equality. By accepting the null hypothesis (the values do not differ), all laboratory values collected preoperatively (n = 30) are assumed to be comparable to the population, and thus a group-specific comparison between the entire set of initial preoperative laboratory values with the recent specific after surgery laboratory values of each group was possible.

## Results

### Histology

#### Inflammatory response

Significant differences in the inflammatory reaction were not found, neither in the lymph nodes nor at the implant site of all study groups in comparison to the control group with the Mann–Whitney-U-test. Statistical testing of semiquantitative histologically determined inflammatory changes by means of the Kruskal–Wallis test showed no p-values below the established significance level of 5%, indicating that there are no significant differences in inflammatory changes between groups. For this reason, there was no need for further statistical testing of individual groups using the Mann–Whitney test for two independent samples.

In all groups, some individual animals exhibited inflammatory reactions in the regional lymph nodes such as inflammatory infiltrates, sinus histiocytosis and follicular hyperplasia. Inflammatory infiltrates consisting of neutrophils or eosinophils were rarely found. Follicular hyperplasia was detectable in all examined localizations and in each group including the control group. At the implant site, a chronic inflammatory reaction was present in a few individual animals as well. Infiltrates consisted of lymphocytes, plasma cells, macrophages and occasional multinucleated giant cells, varying from diffuse to focal extensive infiltrates. Occasionally, focal fibrosis and calcification were also observed.

#### Iron deposits

Analysis of iron deposits within the various organs served as a second assessment criterion. In iliofemoral lymph nodes, spleen, liver, heart, lungs, kidneys and brain, no significant iron deposits could be found (Mann–Whitney-U *p* > 0.05), whereas the iron deposits significantly increased in the tissue adjacent to the implant of all implant groups compared to the control group (Mann–Whitney-U *p* < 0.05). In the short-term group with an implant density of 1.4 g/cm^3^, there were significantly increased amounts of iron deposits in the popliteal lymph nodes on both sides (Mann–Whitney-U, left site test value 28.50 and *p* = 0.013, right site test value 29.50 and *p* = 0.016). For all other groups, such an increase could not be detected.

**Figure 2:**
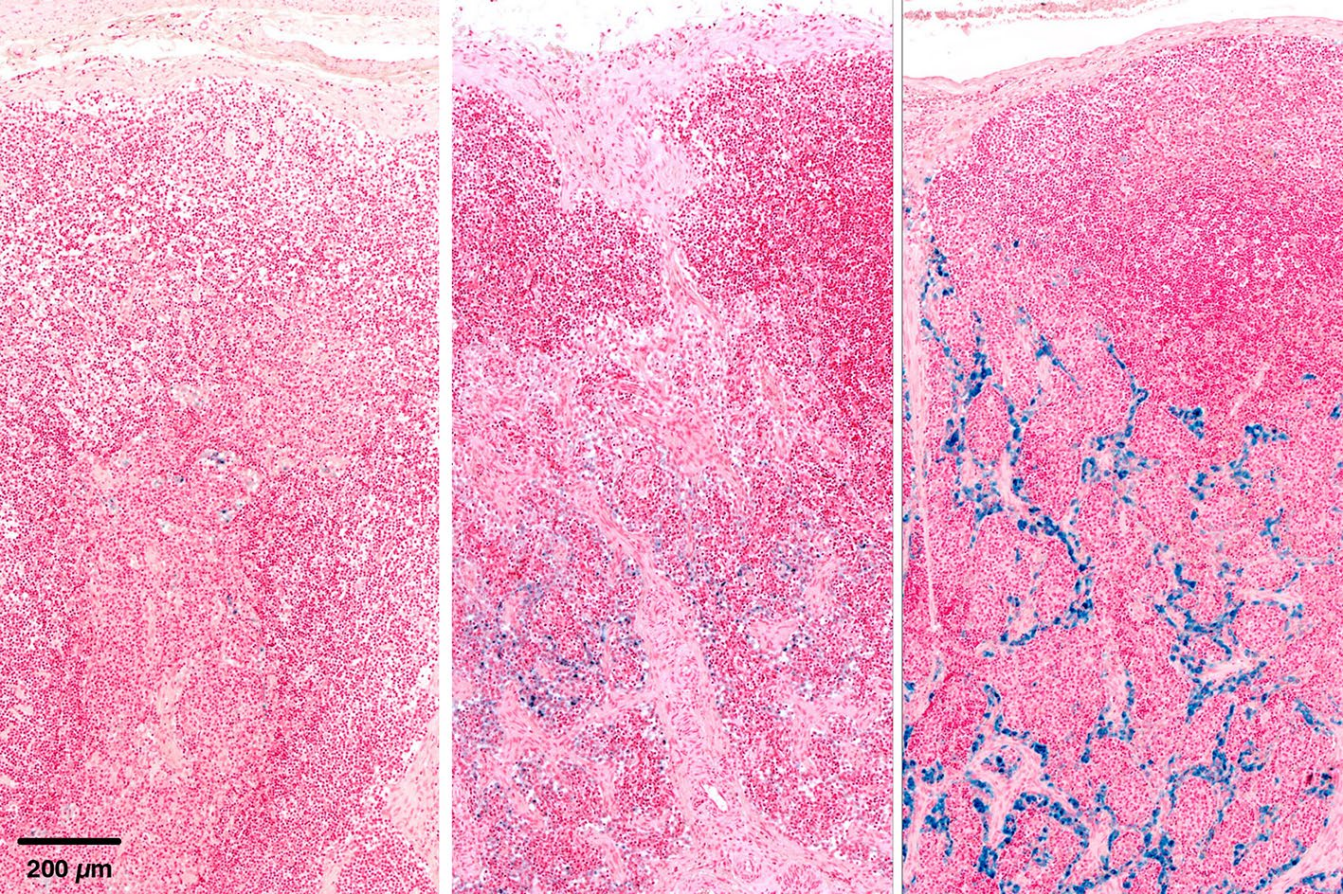
Iron-laden macrophages in the fibrous tissue adjacent to the implant. Adjacent to the macrophages are a low number of lymphocyte has accumulated around the small vessels. HE (left) and Turnbull blue staining (right).

**Figure 3:**
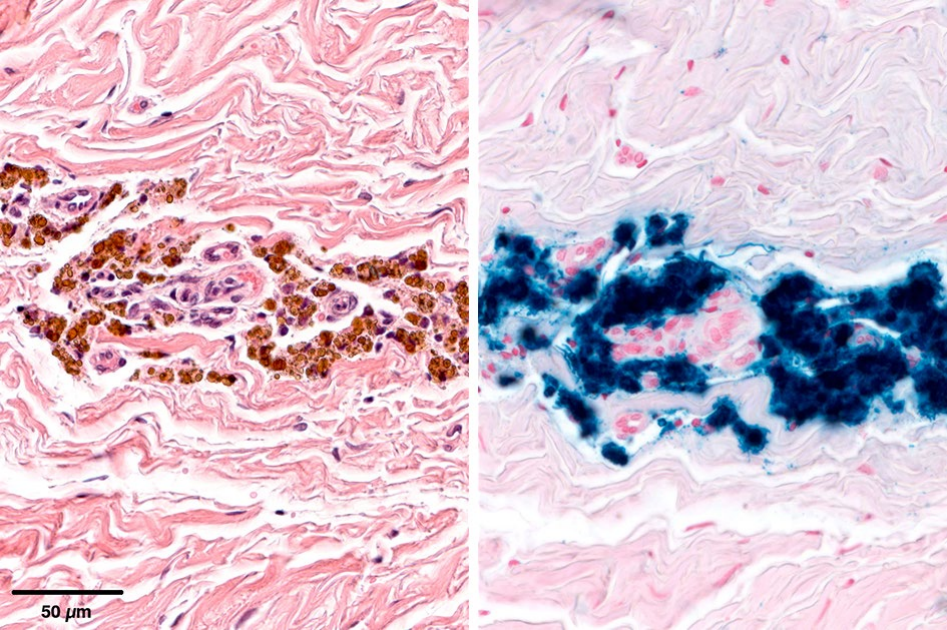
Demonstration of different grades of iron deposition in local lymph nodes. The iron is stored as hemosiderin within macrophages laying in the neighborhood of sinuses. Mild (left), moderate (middle) and severe (right) grade of deposition. Turnbull blue staining.

**Figure 4:**
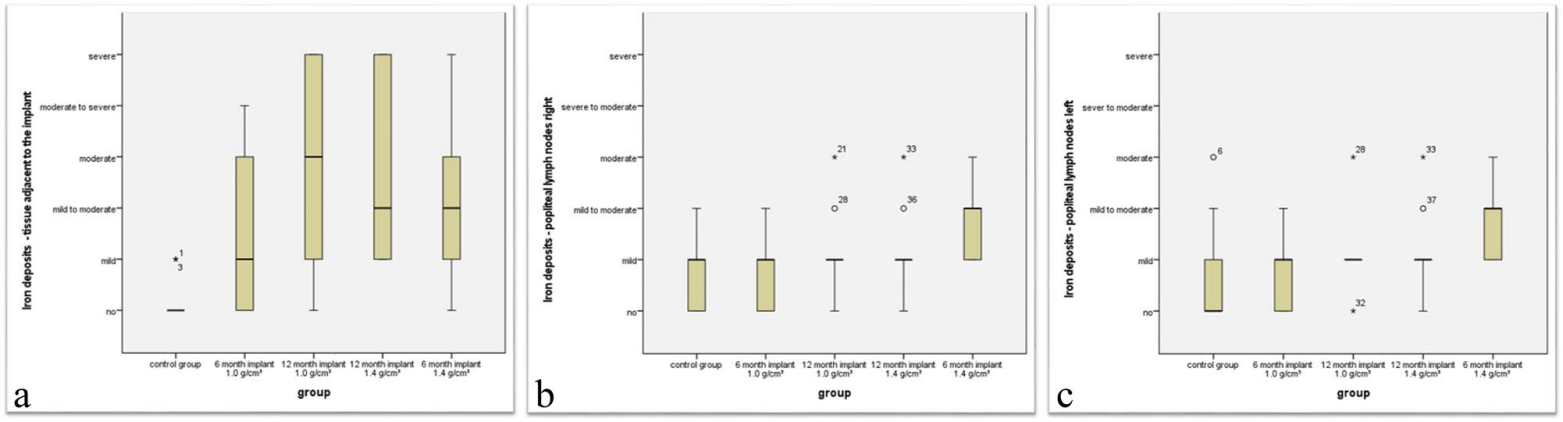
The figure demonstrates the iron deposits in the area around the implants as recorded with a semiquantitative evaluation. All implant groups showed significantly more iron deposits than the control group. There were no significant differences within the implant groups (**a**). The 6-month group 1.4 showed significantly increased iron deposition in the politeal lymph nodes on the right (**b**) and left site (**c**).

Where iron pigment was detected, it was predominantly detectable as intracytoplasmatic granular to coarse deposits inside phagocytic cells. The pigment was yellowish to brown in H&E and stained positive in the iron staining. In sections of the tissue adjacent to the implant, iron was found intracellularly in histiocytes and partly in multinucleated giant cells being scattered in the connective tissue or being focally accumulated (Fig. 2). In animals with a high degree of iron deposition, pigment was not only found intracytoplasmatically in histiocytes but also arranged as fine granular deposits in the connective tissue. In lymph nodes, iron was found in iron-laden macrophages within the sinus (Fig. 3). According to the amount of iron-positive cells per section, the level of iron deposition in organs and lymph nodes allowed the following semi quantitative graduation: no iron deposit, mild iron deposit, moderate iron deposit and severe iron deposit (Fig. 4).

### Laboratory results

#### Iron

We studied whether differences in serum iron levels occurred between the experimental groups after 22, 28 or 42 weeks after surgery. Only 22 weeks after surgery, serum iron levels were significantly increased in the 6-month implant group 1.4 g/cm^3^ (mean 30.58 µmol/l, SD 4.93) compared to the control group. Even in comparison with the average of all preoperative iron levels, no significant correlation to the individual test groups was seen. In determining the Spearman´s rank correlation coefficient, the entire implant group 1.4 g/cm^3^ displayed a correlation of + 0.289 between the time of laboratory testing and the measured serum iron levels.

#### Ferritin

A significant increase in ferritin levels was evident when comparing the population of all preoperative ferritin levels (mean 190.55 ng/ml, SD 238.24) with the results of the 6 month group 1.4 g/cm^3^ at 28 weeks (mean 267.99 ng/ml, SD 181.70) and at 52 weeks (mean 317.65 ng/ml, SD 208.51). An equally increased ferritin level compared to the pre-operative values was only found in the 12 month group of 1.0 g/cm^3^ after 52 weeks (mean 280.66 ng/ml, SD108.52). When calculating the Spearman´s rank correlation coefficient for the implant group 1.4 g/cm^3^, a correlation of + 0.238 between the time of laboratory testing and measured serum ferritin levels arises.

#### Phosphorus

Similar to the findings for ferritin, a significant increase in serum phosphorus levels was found in the study group 1.4 g/cm^3^ compared to the population of all preoperative phosphorus levels at 6 month group at 28 weeks (2.729 mmol/l, SD 0.659 and 52 weeks (mean 2.536 mmol/l, SD 0.314). Also, in the group of 1.0 g/cm^3^ the phosphorus level was significantly increased after 52 weeks (mean 2.783 mmol/l, SD 0.491) in relation to the preoperative values (mean 2.070 mmol/l, SD 0.398). In determining the correlation coefficient, a positive correlation of + 0.405 was displayed between the time of laboratory testing and measured serum phosphorus level for the implant group 1.4 g/cm^3^.

#### Alkaline phosphatase

No significant changes or correlations for serum levels of alkaline phosphatase were observed.

#### Haptoglobin

Longitudinal analysis of the implant group 1.4 g/cm^3^ unfolded a slightly negative correlation coefficient with a value of—0.143 between test time and haptoglobin levels.

#### Leukocytes

No significant changes in leukocyte levels were shown at any time.

#### Blood differential count

No significant changes were seen for any of the recorded parameters (neutrophilic granulocytes, eosinophilic granulocytes, basophilic granulocytes, lymphocytes, monocytes) at any time.

#### Platelets

Platelet counts were not altered in the course of the experiment.

#### Erythrocytes

Only after 28 weeks, a significant increase in erythrocyte count (mean 11.46 × 10^6^/µl, SD 1.44 × 10^6^) occurred in the 6 month group 1.4 g/cm^3^ compared to the control group (mean10.58 × 10^6^/µl, SD 1.16).

When calculating the Spearman´s rank correlation coefficient for implant group 1.4 g/cm^3^, a positive correlation of + 0.273 was found between the time of laboratory testing and the measured number of erythrocytes.

#### Hemoglobin

A positive correlation of + 0.301 between time of testing and specific laboratory hemoglobin values was verified by correlation coefficient determination concerning the implant Group 1.4 g/cm^3^.

A summarized overview of histological and laboratory results is shown in Table 1.

**Table 1:**
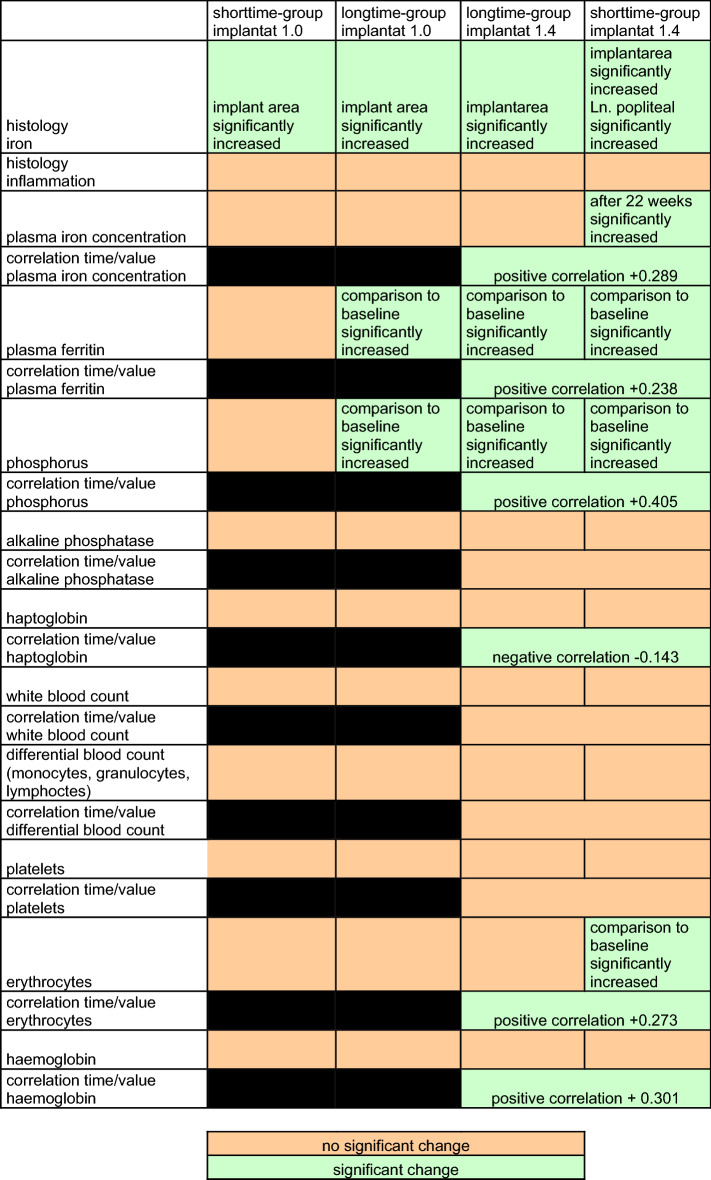
Summarized overview of histological and laboratory results.

## Discussion

To answer the question whether implantation of newly developed iron alloys as bone substitutes causes pathologic deposition in tissues and organs, liver, spleen, kidneys, heart, brain, supraregional lymph nodes and implant site were tested. In order to monitor systemic responses, laboratory tests were conducted to screen for inflammatory markers and iron metabolism parameters.

Statistical analysis of inflammatory organ changes did not reveal any significant implant-related changes compared to controls. In contrast to these findings, iron deposition around the implant site was significantly elevated in all implant groups. In addition, popliteal lymph nodes were affected in the 1.4 g/cm^3^ group, though no other organs were impaired.

A number of studies, particularly in the context of haemochromatosis, have shown deposition of iron in liver, pancreas, heart muscle, kidneys, skin and joints associated with excessive storage^37^. Against this background, an absence of more severe iron deposits in the above-mentioned organs is clear evidence of only minor deposition during the degradation of the implants.

There are only a few studies on biodegradable porous iron-based implant materials in general^31–33^ and only one in-vivo study^34^. The latter does not observe matters of tissue reaction and inflammation in ex-vivo investigations. However, a study performed by Ulum et al. focusses on the cellular response and iron ion levels in the vicinity of implanted Fe, 316L and Fe-HA, Fe-TCP and Fe-BCP composites implanted into the periosteum of the medio proximal region of sheep radial bones^38^. A less pronounced swelling of the implant site indicates a less pronounced inflammatory response for pure Fe compared to 316L. This is confirmed by the blood cell count performed for pure Fe implants that shows normal numbers for erythrocytes (indicating low toxicity), lower number of leucocytes compared to 316L (indicating lower rejection) and a lower neutrophil to lymphocyte ration than for 316L (indicating a lower cellular stress level). Giant cells were found in the vicinity of all investigated materials, however, there was less granular tissue found around pure iron implants compared to 316L implants. Finally, more iron ions were released from pure Fe during the first 2 weeks after implantation than from all other investigated materials, but the Fe blood plasma level remained within normal range in all sheep.

The question of local toxicity or inflammatory reactions and of possible iron deposition after implantation of a biodegradable iron implant in the form of a stent was investigated by Peuster et al. who came to similar results. In their study, biocorrodible stents consisting of 99.5 wt.% iron were implanted into the descending aorta of minipigs^39^, while an additional non-corrodible stent was positioned by catheter intervention. Throughout the experiment, leukocytes and serum iron levels were constantly normal. Animals were euthanized at various time points after stent implantation to perform histological evaluation of lung, heart, spleen, liver and kidneys. In accordance with our results, no increased iron deposition was shown in these organs, but a slight increase in the number of extracellular iron deposits and iron- laden macrophages in the local lymph nodes was found^39^. This effect was observed for the first time one month after stent implantation and for all time points thereafter.

In another paper by the same group published in 2001, similar results were presented for an iron-based degradable stent implanted in the descending aorta of rabbits. In addition to regular angiography to assess the patency of the aorta, histopathological examination of spleen, liver, kidneys, lungs and heart, and the implant-related para-aortic tissue were carried out^22^. In these analyses, few iron deposits and small lymphocytic infiltrations in the para-aortic tissue and the kidneys were detected, while no changes were found in the spleen, liver, lung or heart. Analysis of the vessels supplied with the stent matched our own results as iron-laden macrophages and multinucleated giant cells were observed.

A study on nitrided iron stents implanted in the right iliac arteries of pigs by Feng et al. showed a constantly increased inflammatory response (ranging between moderate and abundant) in the first 6 months after implantation^40^. With advancing degradation of the iron struts, a decreased number of inflammatory cells was found at 12 months after implantation despite a severe accumulation of corrosion products in the tissue. Lin et al. produced similar results for similar pure and nitrided iron stents coated with drug-eluting PDLLA implanted in the abdominal aorta of New Zealand whit rabbits^41^. Mild inflammatory responses were found histopathologically throughout the 13-month implantation period despite the almost complete conversion of the iron to degradation products. However, no infiltration of inflammatory cells like neutrophils, lymphocytes or eosinophils was detectable at any of the time points investigated. Qi et al.^42^ also observed no signs of severe and prolonged inflammatory response for PLA-coated pure iron stents implanted into the abdominal aorta of rabbits by.

Established benchmark values for laboratory data in veterinary medicine are typically based on only a small number of studies and many parameters have not even been sufficiently studied yet. In this sense, current literature does not provide reference values for serum ferritin. Comparison with the control group and longitudinal assessment of ferritin levels during the experiment are extremely important and helpful. in 2011, a publication illustrated for the first time the association between systemic inflammation and decrease in serum iron concentration in sheep^43^.

Veterinary medicine literature reports varying reference values for serum iron levels in sheep, ranging from 18.8 to 34.3 µmol/L^44^, 18 to 48 µmol/L^45^ or 29 to 40 µmol/L^46^. Considering the most accepted reference value 18 to 48 µmol/L, none of the measured serum iron values in our study is outside the defined reference range, which suggests that no significant iron burden occurred^45^. An animal experiment on pigs demonstrated no significant differences in iron levels initially as well as one day or 360 days after implantation of a biodegradable stent^39^. Matching our own results, the reference range for serum iron was not exceeded in any of the animals. This is of particular importance, since the implant used by Peuster et al. contained a much smaller amount of iron (200 mg) compared to the approximately 1.2 g or 1.6 g used in our study^39^. As 1.6% phosphorus is contained in the iron alloys, serum levels of phosphorus were also determined by chemical analysis. The level of phosphorus, which acts as an essential trace element in the sheep organism, has been studied in various publications. Serum standard values of 1.0 to 2.6 mmol/L^47^, 1.2 to 2.3 mmol/L^44^, 1.23 to 1.98 mmol/L^48^ or 1.2 to 2.5 mmol/L are defined for sheep^46^. Compared to the reference range by Pernthaner et al. of 1.0 to 2.6 mmol/L, only one animal tested at the beginning of the trial showed phosphorous levels outside the reference range^47^.

Standard values of alkaline phosphatase in sheep range between 46 to 395 U/L^44^; 44 to 355 U/L^47^, 45 to 208 U/L^48^ or 4 to 175 U/L^49^. None of the measured values within the study period exceeded Dias´ et al. reference range of 4 to 175 U/L^49^.

Serum levels of acute phase proteins (APP) change during the course of infection, inflammation, tissue injury, neoplastic processes or stress^50,51^, stimulated by proinflammatory cytokines such as IL-6 or TNF-α^52^.

Various studies have shown that APP vary greatly in their reactions between different species^51,53,54^. The largest increases in acute-phase response were found for haptoglobin, C-reactive protein on the other hand seems to be of no importance in small ruminants^51,53,54^. This is confirmed by a fast and significant increase in haptoglobin levels subsequent to sheep`s infection with Corynebacterium pseudotuberculosis. Within four weeks values hit reference range, while serum peak values (factor 17) were seen until 1–2 weeks after infection^54^.Therefore, haptoglobin appears to be suitable to detect acute infections or to assess acute systemic immune responses in sheep. The reference haptoglobin serum level for mature ruminants is reported to be below 2 mg/dL^54^, while in young sheep a range of 6 to 12 mg/dL was shown^55^.

Upon acceptance of the reference value by Eckersall et al. only three measurements exceeded 2 mg/dL^54^. In summary, there is no evidence of a significant acute phase reaction in response to the degradable bone substitute material implantation.

Summarizing blood count analyses, no changes were seen in the number of leukocytes or differential counts and therefore chronic inflammatory changes in the experimental animals are highly unlikely.

### Limitations

In our recently published study we were able to show that the implant did not fully degrade within the expected period of 2 years. As a consequence, there may not have been enough degradation products to reach toxic levels. In addition, the effect of ionized iron species must be questioned.

Initial loss of laboratory values due to hemolysis in the control group and the short-term implant group 1.0 g/cm^3^ is a systematic problem and imparts an even higher significance to assess laboratory values longitudinally within each group. By developing a statistical correction model workaround, we were able to compensate for this limitation. Thereby, all available preoperative laboratory values were defined as a population by positive statistical testing and could therefore be used for comparison within the experimental groups.

To enable a more complete evaluation of the systemic iron balance, subsequent studies potentially should measure transferrin values and transferrin saturation in addition to serum iron and ferritin. Potentially even liver biopsy may be performed in order to detect iron overload to rule out implant independent increases in serum iron and ferritin values^56^.

Baumgartner kept 1-year old sheep on a farm and measured phosphorus levels at regular intervals over a year to determine possible seasonal variations^57^. In his experiment, a significant difference in phosphorus levels was described showing higher values in summer and lower values in winter. However, all values were within the reference range identified by Pernthaner et al.^47^. Therefore, dietary seasonal variations for phosphorus content must be considered as a possible cause for the increase in value in our study.

After 6 and 12 months, we identified many solid degradation products in the microscopical evaluation. Particles were identified inside the implant and in the surrounding soft tissue. Also, macrophages with phagocytosed particles were detected. There are only few in-vivo studies on degradation behavior and the corresponding tissue reaction so far and all of them only consider blood vessel wall tissues^40–42,58^. All of these studies observed a certain degree of prolonged inflammatory response, but none addresses a possible correlation between solid degradation products and inflammatory reaction in detail. This matter should therefore be of intensified interest for both vascular and bone tissues in future studies.

## Conclusion

Large bone defects represent a relevant medical problem within musculoskeletal surgery. Therefore, the development of biocompatible bone substitute materials with adequate material properties is a challenge of biomedical research. To address this problem the idea of temporarily replacing these defects by an iron-based biodegradable implant that allows bone ingrowth and degrades over a defined period of time was developed.

Aim of this part of the present study was to determine toxicity during degradation of this iron based implant focusing on inflammatory response, deposition of iron particles in organs and iron metabolism in mammals. Histopathological sections of parenchymatous organs, brain, implant site and local lymph nodes showed no significant inflammatory changes compared to the control group. Increased deposition of iron products were found exclusively in the implant adjacent soft tissue and partially in the popliteal lymph node. Blood and differential blood count values did not indicate an implant related acute or chronic inflammatory response. Minor increases in values of ferritin and phosphorus are detectable in the implant groups compared to preoperative values, but none exceeded the sheep-specific reference ranges. In conclusion, no significant implant-related changes were seen in the laboratory tests. Overall, the iron-based alloy with 1.6% phosphorus qualifies as a useful degradable bone substitute material.

In our recently published study we were able to show that the planned degradation of the implant could not be achieved within a period of about 2 years. If iron is to be retained as the basic material for a degradable bone implant, a method must be developed to drastically reduce the metal content. Alternatively, the alloy must be changed or the material iron as the basis of the alloy must be questioned.
